# Annotations

**Published:** 1910-04-02

**Authors:** 


### Annotations.

### Professor R. Kutner, C.V.O.

Post-graduate students will note with pleasure that H.M. the King has been graciously pleased to confer upon Professor R. Kutner, the Director of the Kaiserin Friedrich Haus, Luizenplatz, Berlin, the honorary Commandership of the Victorian Order, and will, we feel sure, join with us in cordially congratulating the recipient upon the honour. Professor Kutner's name is well known in connection with the promotion of graduate study in medicine. To him, more than to anybody else, Germany owes her magnificent advance in this matter, which we in England can only envy, though we hope to realise similar ideals in time. The Kaiserin Friedrich Haus is the post-graduate centre of the universe, and Professor Kutner is always ready to place his experience and advice at the disposal of student-practitioners from all parts of the world. At the International Congress of Medicine, held at Budapest last year, he was largely and primarily instrumental in founding the International Committee for Post-graduate Instruction in Medicine, which we are glad to see has gained the official sanction and support of the British Government. His zealous efforts on behalf of graduate education have been recognised in his own country as well as by other nations, and the honour which has now been conferred upon him by King Edward is a fitting and graceful recognition of the value of his endeavours in the interests of British practitioners.

### Coroners and Inquests.

More than a year ago a special Departmental Committee was appointed by the Home Secretary to inquire into the law and practice relating to coroners' courts and inquests, with, later on, the additional instruction to investigate the question of deaths under anæsthetics and the danger arising from the use of flannelette. This Committee has now issued its first report, dealing with the subject of coroners and inquests, and in another part of this issue we give a summary of its recommendations so far as these are of more particular interest to medical men. The further reports on the flannelette and anæsthetic questions will, we understand, be issued very shortly. With the recommendations of the Committee, so far as procedure, the matter of fees to medical witnesses, and inquests generally are concerned, every practitioner must cordially agree. None of them need extended comment here, for we have on more than one occasion advocated the reforms which the Committee now advises, and have in doing so pointed out the strong arguments which favour such reforms. Residents will note with delight that there is at last a chance that the old grievance, that charitable post-mortems were never paid for, will be removed in consequence of the Committee's recommendation. Those who consider that live burial is a dangerous possibility under our present system of death certification will find their convictions strengthened by the grudging admission that "it is no fault of the present law if premature burials do not take place," and we, who hold with the Committee that the negative evidence is distinctly reassuring, can only express our satisfaction at the strong commentary which the Committee has thought fit to make on the present system of death certification—a commentary which is in every way justified and which should draw public attention to the need for reform in this matter. It now remains for the Government to act upon the recommendations put forward in this report, and we sincerely trust that such action will not be unduly delayed. These reforms are not matters of party politics; they are matters of urgent national importance, and the sooner they are attended to the better.

### The Effect of Cold Storage on Vaccine Lymph.

The Medical Officer of the Local Government Board reports that during the past twelve months 400,820 charges of glycerinated calf lymph were issued from the Board's laboratory. In primary vaccination the "case-success" was 99.4 per cent., and the "insertion-success" 95.9 per cent., so that the high quality of the lymph was maintained. Some time ago a preliminary report by Dr. Blaxall and Mr. Fremlin was published on the results of sustained subjection of glycerinated calf lymph to temperatures below freezing-point, and the present report contains further information dealing with lymph kept in cold storage for periods of two years and six months respectively. The two-year-old lymph, when withdrawn from cold storage, was found to be free from extraneous organisms, and when used by public vaccinators in the vaccination of 8,559 cases gave "case" and "insertion" percentage successes of 97.8 and 91.4 respectively. The lymph from six calves in all was used, and in only one of these instances, explicable apart from the cold storage, did the lymph in any degree lose its activity. Since July 1908 the lymphs collected weekly from two calves have been divided into equal portions, one portion being placed in cold store for six months and then issued to public vaccinators, the other portion being issued to public vaccinators without having been previously subjected to a temperature below freezing-point. The communication by Dr. Braxall and Mr. Fremlin contains a comparison of the results of the use of lymph from 54 calves, which were each thus divided into two portions. The samples which had been exposed to a temperature below freezing-point for six months were used for 40,931 vaccinations, and gave a case success of 99.6 per cent., and an insertion success of 96.7 per cent. The portions issued without cold storage at the end of six to eight weeks were used for 44.962 vaccinations, and gave a case success of 99.5 per cent., and an insertion success of 96.5 per cent. Thus the results obtained in both cases are identical. These results have considerable importance, since cold storage will enable a supply of lymph to be prepared and stored to meet any sudden expansion in the demand for lymph that may arise by reason of an outbreak of small-pox.

